# Comprehensive Comparison of Liposomal Bupivacaine with Femoral Nerve Block for Pain Control Following Total Knee Arthroplasty: An Updated Systematic Review and Meta‐Analysis

**DOI:** 10.1111/os.12547

**Published:** 2019-11-25

**Authors:** Yuan Liu, Jun‐feng Zeng, Yi Zeng, Yuan‐gang Wu, Xian‐chao Bao, Bin Shen

**Affiliations:** ^1^ Department of Orthopaedics West China Hospital, Sichuan University Chengdu China

**Keywords:** Femoral nerve block, Functional recovery, Liposomal bupivacaine, Pain control, Total knee arthroplasty

## Abstract

To compare the efficacy of liposomal bupivacaine (LB) and femoral nerve block following total knee arthroplasty, we conducted this systematic review and meta‐analysis. 11 trials with 2,908 patients were included in this study. The pooled data demonstrated that total morphine consumption equivalents during the hospital stay was significantly increased in FNB group. In addition, LB has significantly better outcome in view of the postoperative functional recovery, such as the odds of fall, the incidences of straight leg rise (SLR), the number of patients who can walk independently in the day of surgery,the ambulation distance at POD1, the number of patients discharged at POD1. Consistent with the faster functional recovery, liposomal bupivacaine shortens the length of hospital stay. However, there was no significant difference between LB and FNB in terms of Visual Analogue Score (VAS) during the hospital stay. All in all, liposomal bupivacaine has significantly better outcome in view of the postoperative functional recovery and the length of hospital stay compared with femoral nerve block following the total knee arthroplasty.

## Introduction

Total knee arthroplasty (TKA) is a surgical procedure used to treat patients experiencing end‐stage knee osteoarthritis, and over 700 000 TKA are performed annually in the United States^1^. However, most patients still suffer postoperative pain^2^. Inadequate pain control is usually related to a series of negative effects, such as obstructed functional recovery, extended length of hospital stay, and increased economic load^3^. Optimal postoperative pain management is not only beneficial for early movement and better functional recovery but is also helpful in minimizing the length of stay in hospital and the risk of negative effects^4^. Therefore, there is a need to explore the optimal perioperative pain management for patients undergoing TKA^5^.

So far, at least three types of perioperative pain management following TKA have been explored in the published literature. However, the optimal treatment remains controversial. The first type includes oral or vein opiates, epidural analgesia, and patient‐controlled analgesia (PCA). Although they are effective for use as auxiliary analgesia and pain control, adverse events including nausea, vomiting, respiratory depression, postoperative ileus, urinary retention, and physical dependence have been reported^6^. The second type is peripheral nerve blocks, especially femoral nerve blocks (FNB). FNB are widely used following TKA to manage perioperative pain and decrease morphine dosage^7^. However, FNB are associated with quadriceps weakness, which increases the risk of falls^8^. In addition, quadriceps disability may last beyond 2 days after the cessation of FNB because of femoral nerve paralysis, which influenced the ability to engage in physical exercise after TKA^9^. Third, periarticular injection is considered an alternative technique for pain control following TKA. Although bupivacaine is a familiar and relatively long acting anesthetic agent for local anesthesia, it only lasts for 24 h^10^ In contrast, of liposomal bupivacaine (LB) can last up to 72 h following TKA^11^.

Up to now, there have been eight meta‐analyses comparing the above three types of analgesic methods^4^, ^12^, ^13^, ^14^, ^15^, ^16^, ^17^, ^18^. Two of them compare the efficacy of LB with FNB following TKA^4^, ^17^. However, the conclusions they attain are not completely coherent, and there are two recently published studies not included^19^, ^20^. Furthermore, outcomes relating to postoperative functional recovery, such as falls, range of motion (ROM), and ambulation distance were added to the new meta‐analysis. As a result, we performed this updated review and meta‐analysis to comprehensively compare the efficacy of LB with FNB following TKA. In addition, we discussed the cost of two different methods because more and more published articles have referred to the cost comparison between LB and FNB. We hypothesized that the efficacy of LB was superior to FNB following TKA in terms of postoperative functional recovery.

## Materials and Methods

### *Searching*

Randomized controlled trials (RCT) and cohort studies from electronic databases, including PubMed (since 1966), EMBASE (since 1980), the Cochrane Central Register of Controlled Trials (CENTRAL, since 1980), the Web of Science (since 1966), were systematically searched by two reviewers. “Total knee arthroplasty OR replacement,” “liposomal bupivacaine OR Exparel” and “femoral nerve block” were used as search key words. There was no limitation on language and locality. No grey or unpublished articles were included. Two reviewers independently assessed the studies, and any discrepancies were resolved by consultation with the authors. Because this study was a meta‐analysis analyzing existing articles, ethical approval not necessary.

### *Inclusion Criteria*

Studies were selected if they matched the following criteria in PICOS order: (i) Population, patients experiencing TKA who were demographically alike; (ii) Intervention, periarticular injection of LB; (iii) Control intervention, FNB; (iv) Outcomes, postoperative pain management and functional recovery; and (v) Study design, RCT or retrospective cohort study.

To decrease the influence of mixed pain management, we excluded studies that included other strategies such as adductor canal blockade.

### *Data Screening*

Two reviewers independently screened the information listed on a standard form designed to screen the correlative data from included studies. The data extracted included authors, the year of publication, study type, sample capacity, basic patient information (age, gender, and body mass index), anesthetic methods, follow up, and outcomes. Any disagreements were unified through discussion with other authors. The primary outcome reflected the pain control, including the visual analogue score (VAS) and morphine consumption. Secondary outcomes reflected the functional recovery, including incidences of fall and straight leg rise (SLR), ROM, ambulation time, ambulation distance discharged time, and length of hospital stay (LOS).

### *Validity Evaluation*

Based on the Cochrane Handbook, two reviewers respectively evaluated the methodological quality of included studies. A modified seven‐point JADAD scale was adopted to evaluate the methodological quality for RCT, which consisted of five items, including randomization, concealment of allocation, double blinding, withdrawn, and dropped out^21^, ^22^. The study was regarded as an RCT of high quality if the JADAD score was greater than four points. Two reviewers evaluated the quality of non‐RCT by using the methodological index for non‐randomized studies scale (MINORS), which has a range of scores from 0 to 24^23^. Unified consensus was obtained if there were any different opinions.

### *Statistical Analysis*

We make use of the Review Manager 5.3 software to analyze pooled data. We use the effect value of mean differences (MD) or standard mean differences (SMD) to weigh the effect size for continuous outcomes. We use the effect value of relative risks (RR) to measure the effect size for dichotomous outcomes. We considered the result significantly different when a two‐sided *P*‐value was <0.05.

### *Subgroup Analysis*

We use the *I* ^2^ statistic to test heterogeneity across the studies^21^. We regarded a *P*‐value ≤0.1 or an *I* ^2^ >50% as proof of heterogeneity. A random‐effects model could make the synthesis of results with high heterogeneity more conservative than a fixed‐effects model. Therefore, a random‐effects model was used to eliminate the effect caused by high heterogeneity; a fixed‐effects model was adopted when the heterogeneity was low. We performed subgroup analysis to investigate the source of heterogeneity.

## Results

### *Search Results and Study Characteristics*

**Figure 1:**
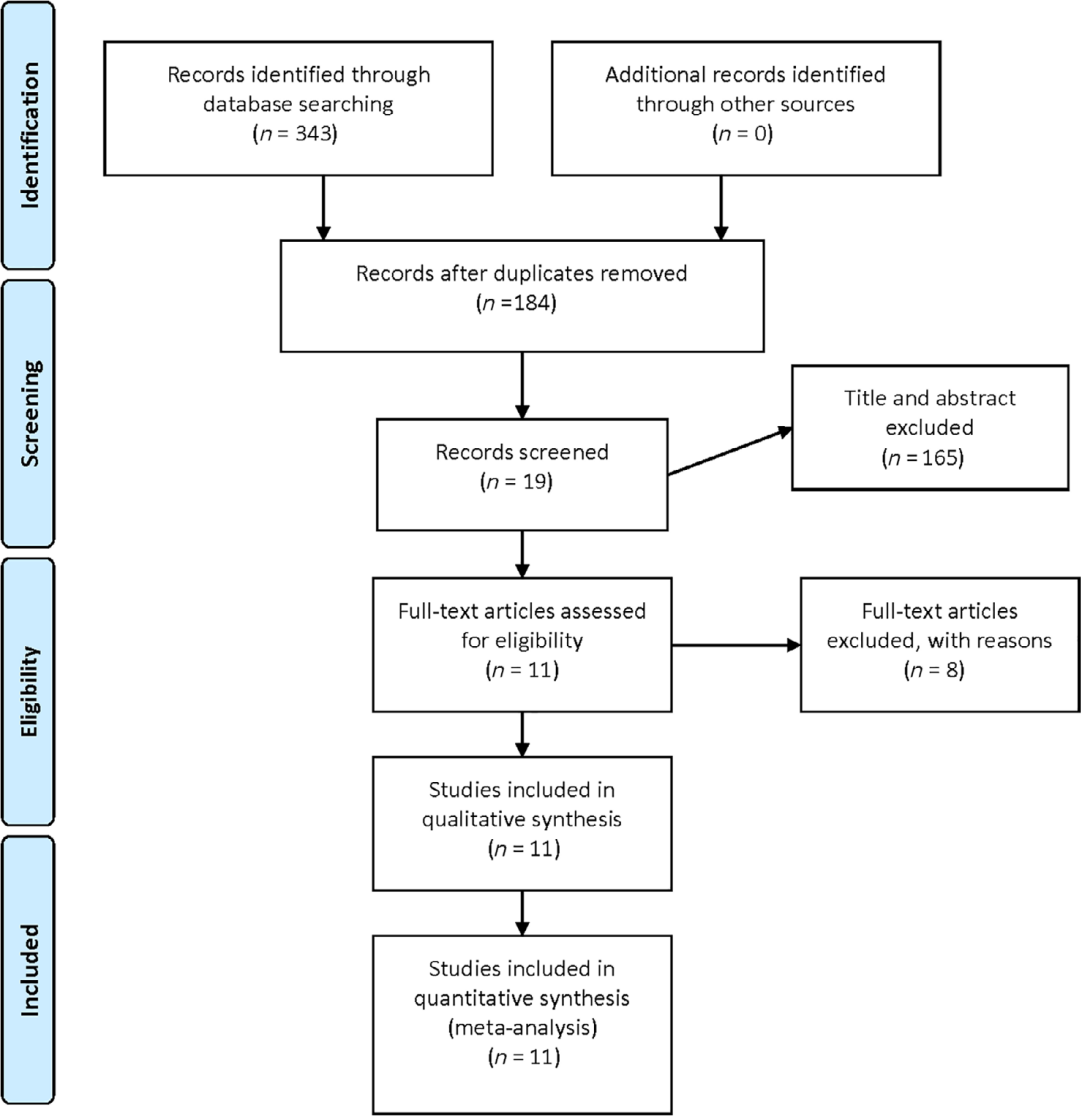
Flow of search results and selection procedure.

A total of 343 relevant articles from electronic databases were identified based on the search strategy. A total of 159 duplicates were removed and 165 studies were excluded after reading the title and abstract. Based on the inclusion and exclusion criteria, 8 studies were excluded by reading the full text. Among them, Phillips *et al*.^24^ added adductor canal block (ACB) to analgesia in the LB group, which could produce bias by lowering the score of the VAS, so we removed it from the meta‐analysis. Kim *et al*.^25^ reported the related outcomes but were excluded because of the repetition of data with another article included in our meta‐analysis. Finally, three RCT^19^, ^26^, ^27^ involving 457 patients and eight non‐RCT^10^, ^20^, ^28^, ^29^, ^30^, ^31^, ^32^, ^33^ involving 2451 patients that compared LB with FNB were included (Fig. 1). The baseline characteristics of each study are presented in Table 1. Five in eleven studies performed power analyses to estimate the sample size to acquire the significant result^19^, ^27^, ^29^, ^30^, ^32^. The length of follow up ranged from 3 days to 1 year.

**Table 1:** The baseline characteristics of the included studies

		FNB/LB								
Studies	SD	Cases	Mean age	Female	BMI	Anesthesia	FNB	Dose of LB	CPM	Follow‐up
Broome (2014)	Non‐RCT	100/100	—	—	—	SA	CFNB	—	Oral morphine	3 days
Cien (2015)	Non‐RCT	65/57	62.6/61.9	41/36	34.5/34.6	GA	SFNB	266 mg LB	Hydrocodone equivalents	1 month
Emerson JR (2016)	Non‐RCT	36/36	64.2/66.9	22/26	34.7/32.5	GA	CFNB	266 mg LB	Morphine equivalents	5 days
Horn (2015)	Non‐RCT	16/16	63.8/66.1	14/14	—	SA	SFNB	266 mg LB	Oral, intravenous, and PCA	2 days
Kirkness (2016)	Non‐RCT	134/134	67.6/67.1	77/74	32.2/32.7	SA/GA	CFNB	266 mg LB	Morphine equivalents	3 days
Marino (2018)	RCT	32/33	64.2/62.3	17/15	33.1/32.6	SA	CFNB	266 mg LB	Oral, intravenous, and PCA	3 days
Sporer 2016	Non‐RCT	325/272	65/63.4	202/168	33.0/33.2	SA	SFNB	266 mg LB	IV hydromorphone, tramadol,tapentadol, morphine andoxycodone acetaminophen	3 days
Surdam (2015)	RCT	40/40	68.4/64.9	21/23	—	SA	SFNB	266 mg LB	IV morphine, Dilaudid, fentanyl and oxycodone	4 weeks
Talmo (2018)	RCT	161/151	62.32/62.01	73/81	30.06/30.71	—	SFNB	266 mg LB	Oral hydromorphone equivalents	1 year
Torres (2017)	Non‐RCT	23/23	66.0/64.4	14/14	—	SA	CFNB	20 cc of LB in 40 cc in 0.9% NS	Morphine equivalents	3 weeks
Yu (2016)	Non‐RCT	583/531	66/65	385/356	32/32	SA/GA	SFNB	20 cc of LB in 40 cc in 0.9%NS	Oral narcotics, intravenousmorphine	3 days

BMI, body mass index; CFNB, continuous femoral nerve block; CPM, concomitant pain management; FNB, femoral nerve block; GA, general anesthesia; LB, liposomal bupivacaine; RCT, randomized controlled study; SA, spine anesthesia; SD, study design; SFNB, single femoral nerve block.

### *Validity Assessment*

The methodological quality of three RCT was evaluated using the modified JADAD scale. For randomization, two were randomized by using sealed envelopes containing the allocation of the group, while the other did not describe the method of randomization. Both refer to the allocation concealment. The patients and surgeons were double blind in two RCT, while not in the other RCT. All three reported on withdrawals and dropouts. The MINORS scale was used to assess the methodological quality for eight non‐RCT with scores varying from 16 to 24. The specific total score of each non‐RCT is recorded in Table 2.

**Table 2:** The quality evaluation of the non‐RCT

Studies	A clearly stated aim	Inclusion of consecutive patients	Prospective data collection	Endpoints appropriate to the aim of the study	Unbiased assessment of the study endpoint	A follow‐up period appropriate to the aims of study	Less than 5% loss to follow‐up	Prospective calculation of the sample size	An adequate control group	Contemporary groups	Baseline equivalence of groups	Adequate statistical analyses	Total score
Broome 2014	2	2	2	2	0	2	2	0	2	0	1	1	16
Cien 2015	2	2	2	2	0	2	2	2	2	0	2	2	20
Emerson JR 2016	2	2	2	2	0	2	2	2	2	1	2	2	21
Horn 2015	2	2	2	2	0	2	0	0	2	0	2	2	16
Kirkness 2016	2	2	2	2	0	2	2	2	2	0	2	2	20
Sporer 2016	2	2	2	2	0	2	2	0	2	0	2	2	18
Torres 2017	2	2	2	2	0	2	2	0	2	0	2	2	18
Yu 2016	2	2	2	2	0	2	2	0	2	1	2	2	19

### *Outcomes of Postoperative Pain Management*

#### *Visual Analogue Score at POD0*

**Figure 2:**
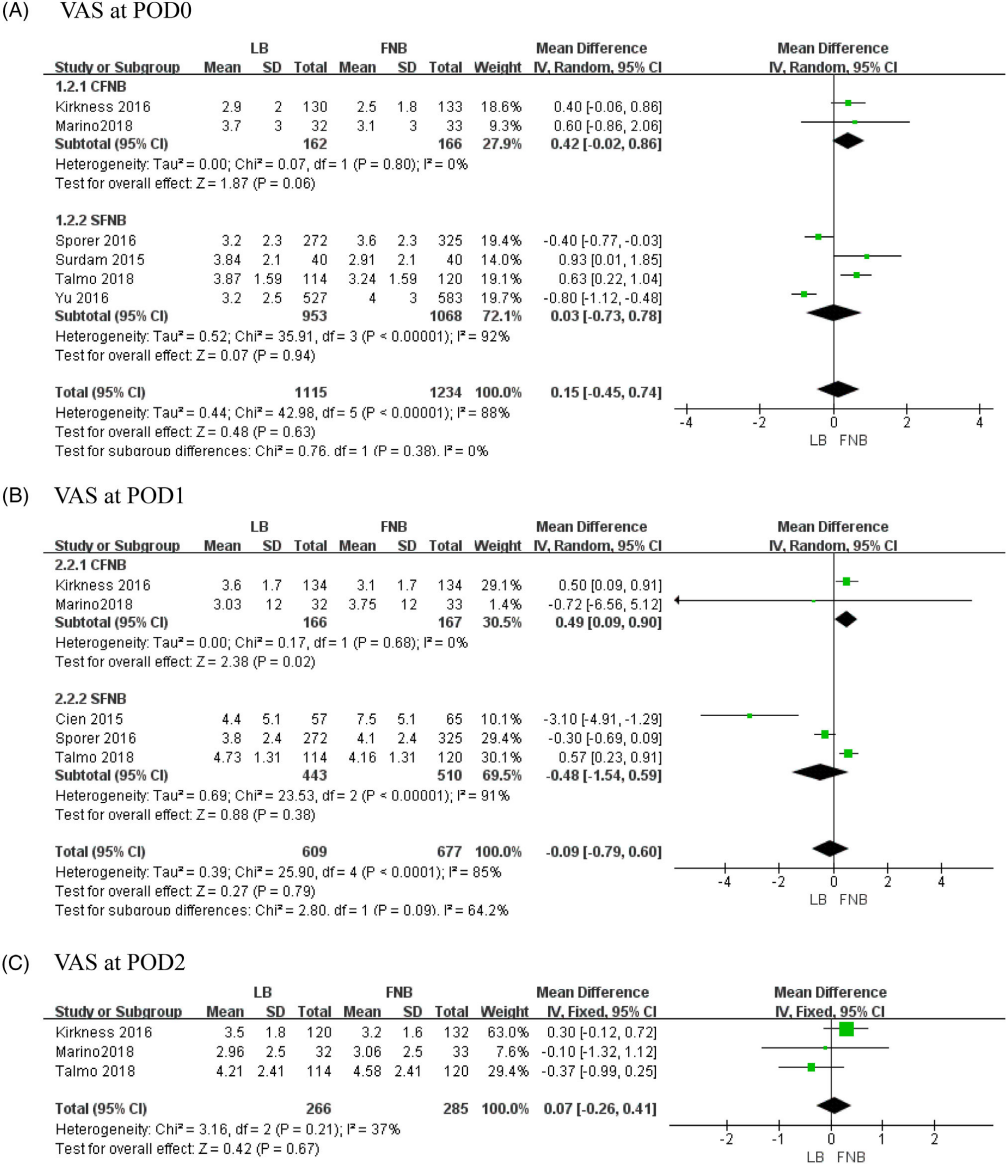
The results of meta‐analysis for outcomes relating to the visual analogue score (VAS): (A) VAS at POD0, (B) VAS at POD1 and (C) VAS at POD2.

Six studies involving 2349 patients referred to the visual analogue score (VAS) on the day of surgery (POD0)^10^, ^19^, ^26^, ^27^, ^32^, ^33^. The final synthesis revealed that the LB is not significantly different from FNB (*MD* = 0.15, 95% *CI*: [−0.45, 0.74], *P* = 0.63; Fig. 2A). Because of the high heterogeneity (χ^2^ = 42.98, *df *= 5, *P* < 0.00001, *I* ^2^ = 88%), a random‐effects model was adopted. The heterogeneity is rooted in the different kinds of FNB according to the results of subgroup analysis (Table 3).

**Table 3:** The results of subgroup analysis

Outcomes	Subgroups	Effect estimate				
		Studies	χ^2^	*MD* and 95% *CI*	*I* ^2^ (%)	*P*
VAS at POD0	CFNB	2	0.07	0.42(−0.02,0.86)	0	0.8
	SFNB	4	35.91	0.03(−0.73,0.78)	92	<0.00001
VAS at POD1	CFNB	2	0.17	0.49(0.09,0.9)	0	0.68
	SFNB	3	23.53	'‐0.48(−1.54,0.59)	91	<0.00001
Total morphine	CFNB	2	0.01	−70.97(−104.11, −37.83)	0	0.93
	SFNB	4	535.92	−36.51(−82.86,9.83)	99	<0.00001
LOS	CFNB	4	1.85	−0.36(−0.53,−0.19)	0	0.6
	SFNB	5	11.82	−0.22(−0.41,−0.03)	66	0.02

#### *Visual Analogue Score at POD1*

Five studies involving 1286 patients recorded the VAS on the first day after surgery (POD1)^10^, ^19^, ^27^, ^29^, ^32^. The final synthesis revealed that LB is not significantly different from FNB (*MD* = −0.09, 95% *CI*: [−0.79, 0.6], *P* = 0.79; Fig. 2B). Because of the high heterogeneity (χ^2^ = 25.9, *df* = 4, *P* < 0.0001, *I* ^2^ = 85%), a random‐effects model was adopted. The heterogeneity is rooted in the different kinds of FNB according to the results of subgroup analysis (Table 3).

#### *Visual Analogue Score at POD2*

Three studies involving 551 patients mentioned the VAS on the second day after surgery (POD2)^19^, ^27^, ^32^. The final synthesis revealed that LB is not significantly different from FNB (*MD* = 0.07, 95% *CI*: [−0.26, 0.41], *P* = 0.67; Fig. 2C). A fixed‐effects model was used because there was no statistical heterogeneity (χ^2^ = 3.16, *df* = 2, *P* = 0.21, *I* ^2^ = 37%).

#### *Total Morphine Consumption*

**Figure 3:**
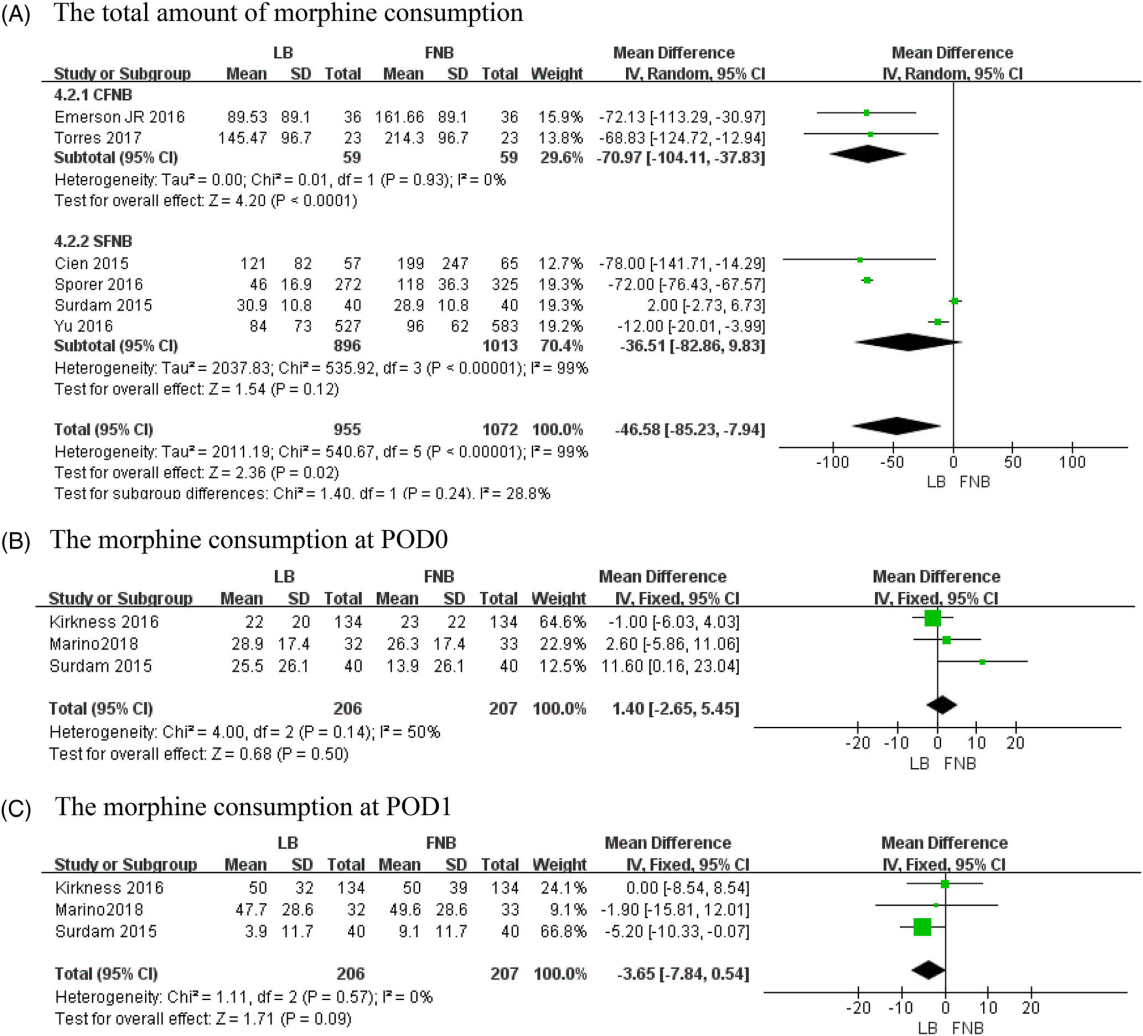
The results of meta‐analysis for outcomes relating to the morphine consumption equivalents: (A) total amount of morphine consumption, (B) morphine consumption at POD0 and (C) morphine consumption at POD1.

Six studies with 2027 patients recorded the amount of morphine consumption during the whole hospital period^10^, ^20^, ^26^, ^29^, ^30^, ^33^. The final synthesis revealed that the FNB group consumed significantly more morphine equivalents (*MD* = −46.58, 95% *CI*: [−85.23, −7.94], *P* = 0.02; Fig. 3). Because of the high heterogeneity (χ^2^ = 540.67, *df *= 5, *P* < 0.00001, *I* ^2^ = 99%), a random‐effects model was applied. The heterogeneity is rooted in the different kinds of FNB according to the results of subgroup analysis (Table 3).

#### *Morphine Consumption at POD0*

Three studies involving 413 patients recorded the amount of morphine consumption on the day of surgery (POD0)^26^, ^27^, ^32^. The final synthesis revealed that LB is not significantly different from FNB (*MD* = 1.4, 95% *CI*: [−2.65, 5.45], *P* = 0.50; Fig. 3). A fixed‐effects model was adopted because there was no statistical heterogeneity (χ^2^ = 4, *df* = 2, *P* = 0.14, *I* ^2^ = 50%).

#### *Morphine Consumption at POD1*

Three studies involving 413 patients mentioned the amount of morphine consumption on the first day after surgery (POD1)^26^, ^27^, ^32^. The final synthesis revealed that LB is not significantly different from FNB (*MD* = −3.65, 95% *CI*: [−7.84, 0.54], *P* = 0.09; Fig. 3). A fixed‐effects model was used because there was no statistical heterogeneity (χ^2^ = 1.11, *df* = 2, *P* = 0.57, *I* ^2^ = 0%).

### *Outcomes of Postoperative Functional Recovery*

#### *Range of Motion*

**Figure 4:**
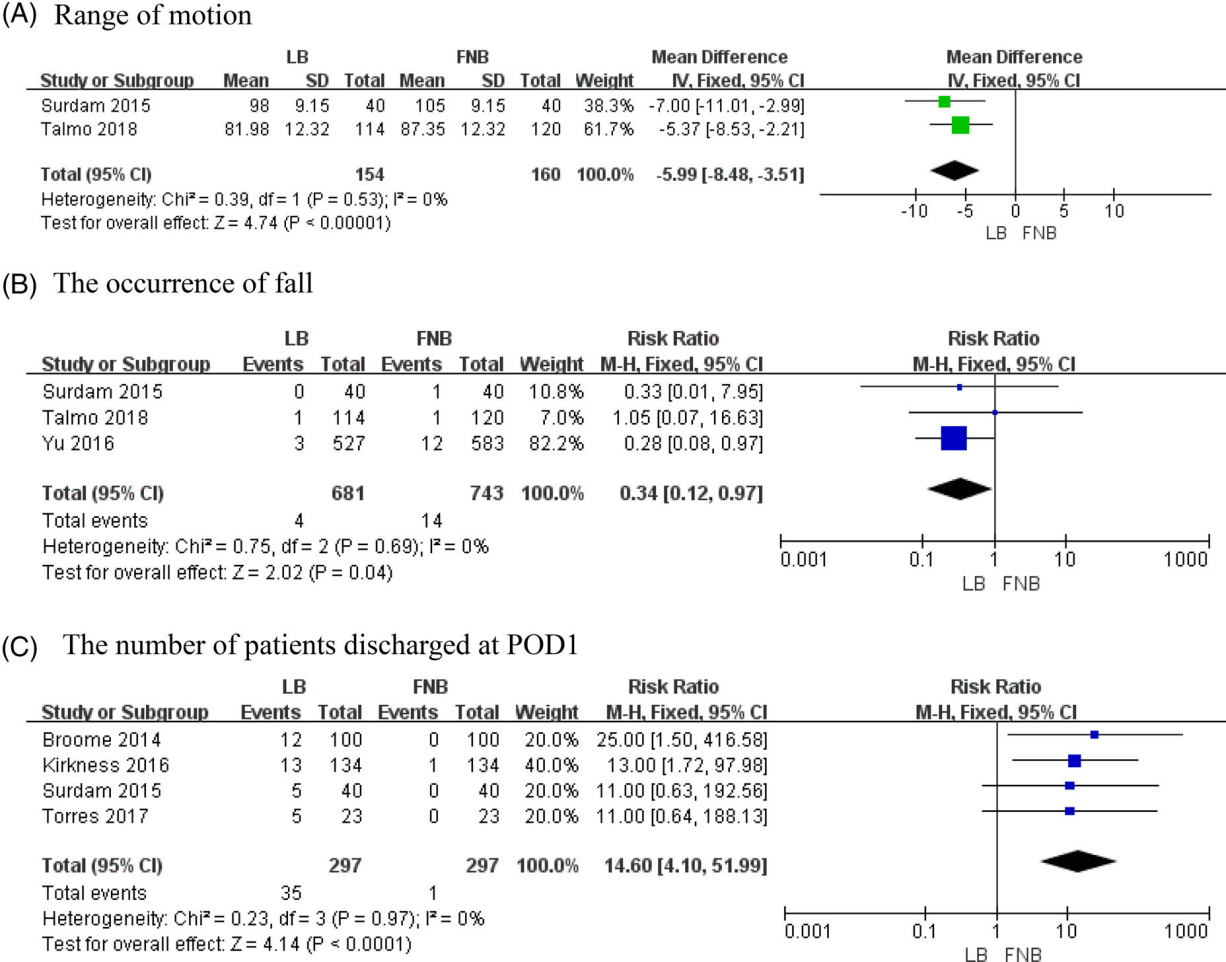
The results of meta‐analysis for outcomes relating to the postoperative functional recovery: (A) range of motion, (B) occurrence of falls and (C) number of patients discharged at POD1.

Two studies involving 314 patients recorded the ROM in two groups^19^, ^26^. The final synthesis revealed that the FNB group may have significantly higher ROM than the LB group (*MD* = −5.99, 95% *CI* [−8.48, −3.51], *P* < 0.00001, Fig. 4A). A fixed‐effects model was selected because there was no statistical heterogeneity (χ^2^ = 0.39, *df* = 1, *P* = 0.53, *I* ^2^ = 0%).

#### *The Occurrence of Falls*

Three studies involving 1424 patients described the occurrence of a fall^19^, ^26^, ^33^. The pooled data revealed that there were significantly more incidences of falls in the FNB group compared with the LB group (*RR* = 0.34, 95% *CI* [0.12, 0.97], *P* = 0.04, Fig. 4B). A fixed‐effects model was selected because there was no statistical heterogeneity (χ^2^ = 0.75, *df* = 2, *P* = 0.69, *I* ^2^ = 0.0%).

#### *Number of Patients Discharged at POD1*

Four studies involving 594 patients described the number of patients discharged on the first day after surgery^20^, ^26^, ^28^, ^32^. The final synthesis revealed that significantly more patients in the LB group were discharged on the first day after surgery compared with the FNB group (*MD* = 14.60, 95% *CI* [4.10, 51.99], *P* < 0.0001, Fig. 4C). A fixed‐effects model was selected because there was no statistical heterogeneity (χ^2^ = 0.23, *df* = 3, *P* = 0.97, *I* ^2^ = 0%).

#### *Occurrence of Straight Leg Rise at POD 0*

Two studies with 298 patients mentioned the occurrence of SLR on the day of surgery^19^, ^26^. The pooled data revealed that the LB group was not significantly different from the FNB group (*RR* = 2.68, 95% *CI* [0.65, 11.01], *P* = 0.17; see appendix). A random‐effects model was adopted because of the high heterogeneity (χ^2^ = 10.79, *df* = 1, *P* = 0.001, *I* ^2^ = 91%).

#### *Number of Patients Walking Independently at POD0*

Two studies involving 348 patients reported on the number of patients who walked independently on the day of surgery^26^, ^32^. The final synthesis revealed that significantly more patients in the LB group walked on the day of surgery compared with the FNB group (*MD* = 6.75, 95% *CI* [3.35, 13.61], *P* < 0.00001; see appendix). A fixed‐effects model was selected because there was no statistical heterogeneity (χ^2^ = 0.04, *df* = 1, *P* = 0.83, *I* ^2^ = 0%).

#### *Ambulation Distance at POD1*

Two studies involving 347 patients recorded the distance patients walked on the first day after surgery^26^, ^32^. The pooled data revealed that patients in the LB group walked significantly farther compared with those in the FNB group (*SMD* = 0.43, 95% *CI* [0.22, 0.65], *P* < 0.0001; see appendix). A fixed‐effects model was selected because there was no statistical heterogeneity (χ^2^ = 0.21, df = 1, *P* = 0.64, *I* ^2^ = 0%).

### *Outcome of the Length of the Hospital Stay*

**Figure 5:**
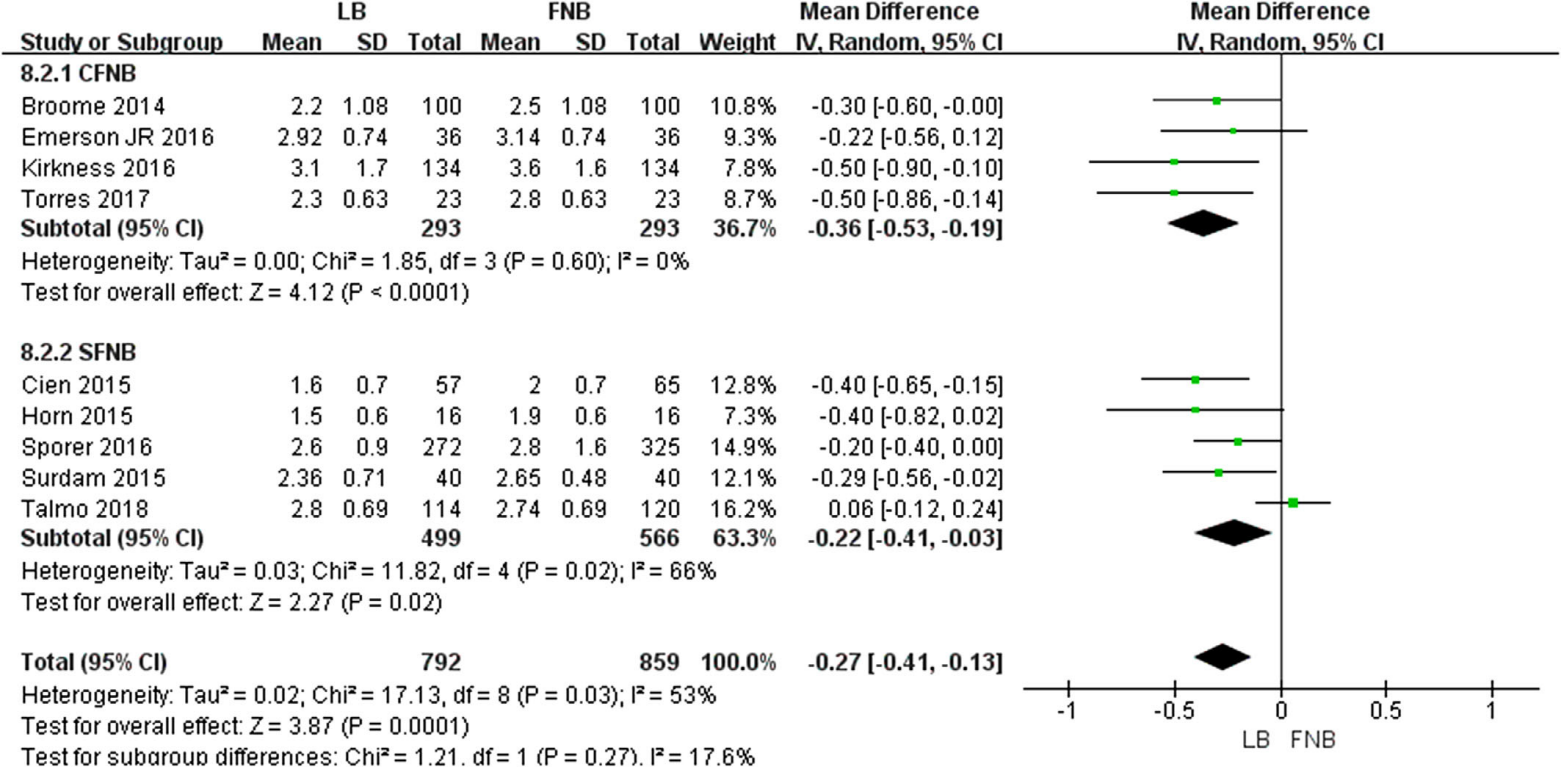
The results of meta‐analysis for outcomes relating to the length of hospital stay.

Nine studies with 1651 patients offer detailed data on the length of hospital stay^10^, ^19^, ^20^, ^26^, ^28^, ^29^, ^30^, ^31^, ^32^. The final synthesis revealed that LB was associated with a significantly shorter length of hospital stay compared with FNB (*MD* = −0.27, 95% *CI* [−0.41, −0.13], *P* = 0.0001, Fig. 5). A random‐effects model was adopted because of the high heterogeneity (χ^2^ = 17.13, *df *= 8, *P* = 0.03, *I* ^2^ = 53%) The heterogeneity is rooted in the different kinds of FNB according to the results of subgroup analysis (Table 3).

## Discussion

Optimal pain management following TKA enables faster functional recovery and reduces postoperative complications and hospital costs^34^, ^35^, ^36^. Accompanied with a steadily rising demand for TKA as the population ages, the obesity rate increases, and surgical techniques advance, both surgeons and patients have an appetite for more satisfactory perioperative pain management, faster functional recovery, and reduced hospital costs^37^, ^38^. Among the current perioperative pain management options, LB has been approved for use by the US FDA, and a series of clinical trials have proven that LB is safe and helpful for pain relief. FNB was used as a general method for postoperative pain control. Two published meta‐analyses compared LB with FNB following TKA, but the conclusions they reached were not totally consistent. Ma *et al*.^4^ investigated one RCT and five non‐RCT and concluded that LB provides similar postoperative pain relief and significantly reduces the consumption of morphine equivalents compared with FNB. However, Liu *et al*.^17^ analyzed two RCT and six non‐RCT and concluded that LB provides a significant beneficial effect over FNB in terms of pain reduction, and decreased the total morphine consumption. In addition, none of them compared the functional recovery in the experimental and control groups. Furthermore, a new RCT^19^ indicated that pain scores were slightly lower in the FNB group in the first 24 hours after TKA compared with LB. It was not clear which method is better in relation to pain relief and functional recovery.

Therefore, we perform this meta‐analysis to judge whether LB is superior to FNB in terms of perioperative pain management and postoperative functional recovery after TKA. To our knowledge, this is the first meta‐analysis that compared the postoperative functional recovery between LB and FNB. The most significant finding of this study was that the LB speeds up the postoperative functional recovery and decreases the length of stay compared with FNB.

In our study, the primary outcomes include the VAS and morphine consumption equivalents, both of which reflect the efficacy of pain relief following the TKA. The pooled data showed that the effectiveness of LB was the same as the FNB for VAS during the hospital stay. However, the total morphine consumption in the LB group was significantly less compared with the FNB group (*MD* = −46.58, 95% *CI*: [−85.23, −7.94], *P* = 0.02), while the morphine consumption at POD0 and POD1 were not significantly different in two groups. This might be explained with two possible reasons. First, the lower the amount of morphine consumption, the shorter the length of hospital stay (*MD* = −0.27, 95% *CI* [−0.41, −0.13], *P* = 0.0001). In this case, we cannot judge which group is superior in terms of pain control. Second, the lower amount of morphine consumption is the result of better efficacy of pain control in the LB group. In this case, we can indirectly conclude that LB is superior in terms of pain control than FNB. The results regarding postoperative VAS and morphine consumption are in line with two previous meta‐analyses. Therefore, we can confidently conclude that LB is at least similar to FNB in regard to the efficacy of pain control following TKA.

We measured the postoperative functional recovery including five items as the second outcome in our study, which was not involved in the two published meta‐analyses. First, we compared the incidence of falls in the two groups. Several studies have previously found that FNB may be associated with the increase of falls. Paauwe *et al*.^39^ conducted a pilot study and found that there was a quantitative quadriceps weakness in 33% of patients with FNB following TKA. Sharma *et al*.^40^ performed a retrospective review of TKA patients after FNB versus no block demonstrated an increased ratio of falls of 1.6 versus 0.4%. This was consistent with our finding (*RR* = 0.34, 95% *CI* [0.12, 0.97], *P* = 0.04). Second, early and safe ambulation is crucial for physical exercise after TKA; thus, we compared the number of patients who walked independently on the day of surgery (POD0) and the ambulation distance at POD1 between LB and FNB groups. The data demonstrated that patients in the LB group walked earlier and ambulated further than those in the FNB group. This was consistent with the finding for falls. Third, to assess the functional recovery of knee joints, we compared the ROM and the number of patients who could perform straight leg rise (SLR) between LB and FNB. We found a trend that patients in the LB group could more easily perform SLR than those in the NB group (Fig. 4). However, the results were significant for the fixed‐effects model but not the random‐effects model. We suspected that the significant outcome might result when a bigger sample size is used. Regarding ROM, two RCT^19^, ^26^ were included in the comparison of ROM. The pooled data showed that the ROM in the FNB group was higher than in the LB group, and this was associated with the time of ROM measurement, which is within 24 hours after surgery. The VAS within 24 hours in these two studies were both significantly higher in the LB group than in the FNB group. Fourth, in consideration of a trend that patients in the LB group can exercise earlier, we compared the number of patients discharged at POD1 and concluded that more patients in the LB group were discharged to home on POD1 than in the FNB group (*MD* = 14.60, 95% *CI* [4.10, 51.99], *P* < 0.0001). Eventually, LB decreased the length of hospital stay (*MD* = −0.22, 95% *CI* [−0.31, −0.13], *P* < 0.00001).

To summarize these results, we explored the reasons for patients in the LB group having a faster functional recovery than those in the FNB. After comparing the detailed information regarding the two analgesia, it was evident that the effective mechanisms of the two methods are completely different. The LB was injected into the surrounding site of the surgery, but the analgesia drugs were injected into the femoral nerve in the FNB group. This produced the side effect of femoral nerve palsy, decreasing the strength of quadriceps, so that the patients in the FNB group had a longer period of functional recovery.

To obtain more convincing pooled data in the comparison of postoperative pain management, future studies should pay more attention to these points. First, it is best to measure the pain score at different time points and to calculate the mean every day to decrease the subjective discrepancies, and to use a unified pain scale such as the VAS to decrease objective discrepancies. Second, to reduce avoidable influences on VAS, it is best to make concomitant pain management consistent. Third, a better functional recovery is an important reflection on better postoperative pain management. Consequently, future studies could add the postoperative functional recovery into the comparison. Fourth, besides the comparison inside the hospital, it is important to evaluate the prospective outcome with a long follow up to explore whether the different analgesia influences the final outcome of TKA.

There are several limitations in our study. First, only two RCT were included; the statistical validity would increase if we included more RCT. Second, four trials had fewer than 50 patients in each group and follow up was not long enough. Third, the heterogeneity of several results is high, which might influence the reliability of our study. Fourth, because of incomplete data, we did not compare the incidence of adverse events such as nausea and vomiting, which is important to reflect the adverse impacts for the two groups.

### *Conclusion*

Liposomal bupivacaine significantly reduced the amount of total morphine consumption and sped up the postoperative functional recovery, reducing the length of hospital stay compared with FNB following TKA.
